# Responders to Platelet-Rich Plasma in Osteoarthritis: A Technical Analysis

**DOI:** 10.1155/2017/7538604

**Published:** 2017-08-20

**Authors:** Christophe Milants, Olivier Bruyère, Jean-François Kaux

**Affiliations:** ^1^Physical Medicine, Rehabilitation and Sports Traumatology Department, SportS2, FIFA Medical Centre of Excellence, University and University Hospital of Liège, Liège, Belgium; ^2^Department of Public Health, Epidemiology and Health Economics, University of Liège, Liège, Belgium; ^3^Department of Sports and Rehabilitation Sciences, University of Liège, Liège, Belgium

## Abstract

**Purpose:**

To evaluate the similarities and differences between the variety of platelet-rich plasma (PRP) formulations, preparation, and uses to try to determine the best responses for the treatment of knee osteoarthritis.

**Materials and Methods:**

A comparison of the outcomes of randomized controlled trials (RCTs) included in the 3 most recent and high-quality meta-analyses to classify the different studies in 2 groups (bad responders group (BRG) and very good responders group (VGRG)).

**Results and Discussion:**

From the 19 RCTs analyzed, 7 trials were included in the VGRG and 4 in the BRG. In VGRG, 1 or 2 injections were performed in 4/7 trials, time between injections was 2 to 3 weeks in 4/5 studies with many injections, volume injected varied from 2.5 to 8 mL, and single spinning technique was used in 5/7 studies. PRP classification was Mishra 4B and PAWP2B*β* in 5/7 studies. The use of PRP with leukocytes is only found in the BRG.

**Conclusion:**

There is a lack of standardization in PRP preparation technique for knee osteoarthritis. However it appears that the use of a single spinning technique, a platelet concentration lower than 5 times the baseline, and avoidance of leukocytes should be preferred.

## 1. Introduction

Knee osteoarthritis (OA) is one of the major causes of pain and physical disability in older adults. Symptomatic knee OA occurs in approximately 13% of people who are aged ≥60 years old [1, 2]. It is a clinical syndrome of joint pain with multifactorial etiopathogenesis that is characterized by the gradual loss of articular cartilage, osteophyte formation, subchondral bone remodeling, and inflammation of the joint [3]. Radiographic knee OA incidence in women ≥55 years was estimated at 2.5% per year [1].

Different methods are used to alleviate the symptoms of patients with knee OA, including analgesics, physical therapy, exercise prescription, and intra-articular injections, such as corticosteroids injection and hyaluronic acid (HA) [4, 5]. Owing to the limited lifespan of joint replacements with implant wear and the associated risk for joint revision, conservative treatment modalities are the central focus in the younger and middle-aged population with cartilage damage and OA of the knee [6].

The management of chondral disease is challenging because of its inherent low healing potential. In fact, the regeneration ability of cartilage is limited due to its isolation from systemic regulation and its lack of vessels and nerves [7, 8].

New studies have focused on modern therapeutic methods that stimulate cartilage healing process and improve the damage, including the use of platelet-rich plasma (PRP), an autologous growth factor treatment [4]. PRP is prepared from autologous blood by centrifugation to obtain a highly concentrated sample of platelets, which is four to five times higher than that of normal blood. The platelets undergo degranulation to release growth factors (GFs) that promote angiogenesis, tissue remodeling (bone, skin, muscle, tendon, etc.), and wound healing [9, 10]. More recently, PRP has been used in the management of knee OA. The importance behind using PRP in cartilage tissue engineering field is that PRP is rich in growth factors, including those that promote proliferation of chondrogenic cells and secretion of cartilaginous matrix [11].

Many papers were published on PRP for knee OA, including a lot of randomized controlled trials (RCTs) and different systematic reviews. Due to the mixed results from controlled studies, the clinical efficacy of PRP in the treatment of knee OA is unclear with shortcomings in the current literature. Indeed, there are variations in the treatment approach including subject, knee and outcome-specific variables, including, but not limited to PRP preparations techniques, platelet count, severity of OA, number of injections, interval/frequency of administration, and a lack of volume standardization [12, 13]. In addition, the use of anticoagulants, activating agents, and separation techniques has varied considerably among studies. Campbell et al. published recently a systematic review of meta-analyses, evaluating platelet-rich plasma injection in the treatment of knee joint cartilage degenerative pathology [14]. They included 3 high-quality meta-analyses. Campbell et al. emphasized that there still remain multiple unanswered questions about the best PRP formulation. Different classification of PRP was proposed by various authors to organize and compare results in the literature but remains probably incomplete considering the number of parameters that can characterize PRP [15].

The objective of this study was to evaluate the similarities and differences between the variety of PRP formulations, preparation, and uses of this techniques and to try to determine characteristics of the PRP which tend to give the best result.

## 2. Methods

In November 2015, Campbell et al. published a systematic review of meta-analyses, evaluating platelet-rich plasma injection in the treatment of knee OA [14]. We selected the studies included in the 3 most recent meta-analyses [16–18] on the topics. One RCT was excluded because it was written in Chinese [19]. Two other RCTs were added, after an updated literature search [20, 21].

We listed the outcomes that were assessed and reported by each study, including Western Ontario and McMaster Universities Osteoarthritis Index (WOMAC), Visual Analog Scale (VAS), International Knee Documentation Committee (IKDC), and Knee Injury and Osteoarthritis Outcome Score (KOOS).

The minimal clinically important improvement (MCII) was defined to help determining whether an observed difference is clinically important. Tubach et al. promoted in 2012 the use of values of MCII, including knee OA, for different outcome criteria [22]. They determined a value of 15 of 100 for absolute improvement or 20% for relative improvement. We used Tubach et al.'s values to classify the different studies in 2 groups depending on the outcomes: bad responders group (BRG < MCII) and very good responders group (VGRG > 2xMCII).

The full texts were thoroughly read to extract features of the PRP used by the various authors from the 2 groups. Features included the number of injections, time between injections, volume of PRP, centrifugation technique, time after blood puncture before injection, anticoagulation, platelet/erythrocytes or red blood cells (RBC)/leukocytes or white blood cells (WBC) concentration, platelet activation, use of NaHCO_3_ tampon, and rehabilitation after infiltration.

We contacted all of the authors by e-mail because of insufficient data in the manuscripts.

To classify the PRP of the different studies, we used the Mishra and PAW classifications [23, 24]. These 2 classifications allow the readers to easily know level of platelets concentration, the presence or absence of WBC, and the activation before injection or not (Table 1).

## 3. Results

A total of 19 articles were selected [20, 21, 25–41]. Five authors (Acosta-Olivo, Jang, Patel, Say, and Vaquerizo) out of 19 (26.3%) gave us more information about the quality of the PRP they used in their study. The outcomes that were assessed and reported by each author are represented in Table 2. The separation between BRG (*N* = 4) and VGRG (*N* = 7) was made from these values. Eight studies were not included in any group.

In 4 out 7 studies of the VGRG, 1 or 2 injections were given, against 3 in all the studies of the BRG. Time between injections tends to be more important in the VGRG (2 to 3 weeks in 4 out of 5 studies with many injections in VGRG in contrast to 1 in BRG). Volume injected varied from 2.5 mL to 8 mL in the VGRG. Good results were objectified with the same technique (Endoret), at very different volumes (from 2.5 to 8 mL). Centrifugation technique was variable. Single spinning technique was the one used most (6/11; 5/7 VGRG, not mentioned in 2/7; 1/4 BRG, not mentioned in 1/4) and this technique appears to give better results than double spinning technique. Endoret was used in 3 of 7 good responders and never in bad responders.

Time after blood puncture before injections was less than 2 hours. Freezing technique was used to conserve the PRP units when other injections were scheduled in the following weeks in 2 to 3 out of 11 studies (*N* = 2/4 in BRG, *N* = 0 to 1/7 in VGRG with many injections as it was not mentioned by Wang et al.). In other studies, blood was extracted each time. Anticoagulation, when mentioned, with citrate (CPDA or sodium citrate) was the only used technique (6/11) and almost always in VGRG (5/7). Activation with CaCl_2_ was used in a lot of studies, from the 2 groups (mentioned in 4/7 studies of VGRG and 3/4 studies of BRG).

Platelet concentration was only available in 7/11 studies. The exact value was measured in only 1 out 11 studies and the mean value in only 1 out 11 studies (while it could have been a very important parameter). Other authors compared the platelet concentrations obtained using different preparation techniques [23, 42, 43]. We reported their results to complete the Mishra and PAW classifications. It is interesting to note that almost all studies from the VGRG were Mishra 4B and PAW P2B*β*. This corresponds to an activated, leukocyte-poor PRP, with a platelet concentration of less than 5x baseline (for Mishra classification), or, more precisely between baseline and 750000 platelets/*μ*L (for PAW classification) [23, 24].

The exact count of RBC and the use of NaHCO_3_ were never available. The use of PRP with WBC is only found in the BRG. There was no standardized protocol of rehabilitation after infiltration. Most of the time (*N* = 6/11, 12/19), relative rest, analgesics, no nonsteroidal anti-inflammatory drugs (NSAIDs), and light activity were recommended, when mentioned.

## 4. Discussion

We observed an important variability of the PRP preparation technique and a major lack of standardization. Furthermore, a lot of information is missing. However, single spinning technique tends to give good results, more precisely the use an activated, leukocyte-poor PRP with a platelet concentration of less than 5x baseline. The use of CaCl_2_ and citrate is frequent. Volume of PRP is inconstant. Leukocyte-rich PRP is only used in the BRG.

Meheux et al. tried to determine similarities and differences in outcomes based on the PRP formulations used in 6 studies [13]. All but one study used leukocyte-poor PRP and showed good outcomes, which did not help comparing the effects of leukocyte-rich PRP versus leukocyte-poor PRP.

We aimed to compare the different formulations of PRPs, after selecting the good and the bad responders, including other features and studies than Meheux et al. November 2012, Tubach et al. promoted the use of values of minimal clinically important improvement (MCII) in reporting the results of trials of different rheumatic diseases, including knee OA, with pain, patient global assessment, physical function, or physician global assessment used as outcome criteria [22]. They determined a value of 15 of 100 for absolute improvement or 20% for relative improvement.

Only 4 trials did not reach this score, we considered them as bad responders. All of the other studies reached this cut-off score (*N* = 15/19). To be more discriminant and to select the studies which results were really the best, we decided to consider as very good responders the trials whose results reached twice this score (Table 3). Seven trials were included in this group.

After comparing the results of our analysis, we could propose that the following criteria should be applied:

(i) To focus more on PRP preparation technique than on volume injected, single spinning technique appears attractive, such as PRGF-Endoret by BTI, which is used in 3 of 6 studies from VGRG. It consists of a simple and rapid protocol, with a single spinning approach during 8 minutes at 1800 rpm. The PRGF obtained by this technique should not contain WBCs and was first defined by Anitua in 1999 [44]. However, this technique appears to be technically imprecise and may lead to irreproducible results because of the pipetting technique, which is operator-dependent and poses a risk of a low platelet collection efficiency, as platelets and leukocytes are found together in the intermediate layer after low spin centrifugation [15]. Filardo et al. compared safety and efficacy of PRP obtained after single (PRGF) or double (PRP) centrifugation [32]. A significant clinical improvement was obtained in the 2 groups, although PRP injections produced more pain and swelling. Methodological changes and lack of reproducible protocol were emphasized by Anitua et al. [45]. Lack of standardization of PRP preparation could have led to changing results in the literature [23]. The only way to have a reproducible PRP is to use an apheresis machine [46]. In addition to this, its use decreases the potential risk of contamination and the unavoidable variability of manual techniques.

(ii) A platelet concentration 3 to 4 times that of whole blood [46] is recommended for treatment of tendinopathies with PRP. Similar concentrations appear to be recommended for osteoarthritis in view of our results. As it was pointed by Delong et al., this most basic measurement is still lacking in the recent publications [23].

(iii) The presence of WBC is still much debated [23, 32, 44, 47]. Braun et al. showed that using PRP rich in leukocytes and RBC resulted in significant synoviocytes cell death and proinflammatory mediator productions [48]. Some authors prefer avoiding WBC in PRP because of the potential deleterious effect of protease and reactive oxygen released from WBC. Others promote the preventive effect of cytokines and enzymes against infection [32]. A preclinical study highlighted that, in a normal equine tendon, leukocyte-riche PRP contributes to inflammatory cytokine production, even if isolated values of IL-1B and TNF-*α* were increased compared to a leukocyte-poor PRP [47]. In our opinion, RBC and WBC should be avoided in PRP preparations. Our opinion is not shared by all. A clinical study compared the intra-articular injection of PRGF, which is a PRP obtained after a single centrifugation and which contains no WBC versus PRP obtained after double centrifugation which contain WBC. Both treatment groups presented a similar statistically significant improvement even if the comparative analysis showed more swelling and pain after PRP injection [32]. Furthermore, Mariani et al. showed no upregulation of proinflammatory mediators after leukocyte-rich injection for knee OA in synovial fluid or plasma [49].

(iv) As for tendinopathy treatment, it is necessary to limit the number of injections to avoid side effects and increase in the price of the treatment [50]. Few authors compared the effect of one versus multiple PRP injection for knee OA. Discordant results are available in the literature. Patel et al. compared the effect of a single injection to 2 injections of PRP 3 weeks apart. Short-term results (6 weeks) were similar between the two groups. A longer follow-up (3 to 6 months) showed that the effect tends to taper over time in the two groups [26]. More recent studies demonstrated beneficial effects of multiple injections. Görmeli et al. showed better results after 3 injections against a single injection for early knee OA after 6 months. For advanced OA, no difference was found [51]. In our daily practice, we realize a total number of 2 injections, as suggested by Kavadar et al. who compared the effects of a single injection, 2 injections, or 3 injections of PRP in moderate knee OA [52]. Future comparative multicentric RCTs on this topic with long term follow-up period should be realized to bring out the most effective injection number.

(v) Injection interval is of 2 to 3 weeks.

(vi) Freezing technique could lead to damage of the PRP product [45]. Sonker and Dubey showed a steep fall in growth factor levels after 5 days of storage after a preparation applying the double freeze thaw technique for the growth factor release [53]. This appears confirmed by our study, because only studies from the BRG (2/4) used this technique to preserve the PRP for further injections. Even if a good clinical efficacy is obtained in studies with PRP stored by freezing [32], it is possible that even better results were obtained with nonfrozen PRP. Note that one author of the VGRG did not mention whether this technique was used or not [37]. The use of an anticoagulant is necessary, citrate is recommended as it preserves platelet reactivity, compared to EDTA and heparin [46]. No difference appears between sodium citrate and citrate dextrose [54].

Even if some authors gave us an answer, some features were not available, because they were not measured and are missing in our comparison table. Platelet concentration, presence or absence of red or white blood cells and its concentration, and use of tampon NaHCO_3_ were rarely, if ever mentioned. This information should be mentioned in future studies to help determine which PRPs tend to give the best results for osteoarthritis, preferably using a peer reviewed classification system, such as PAW and Mishra classification.

In the literature, other recommendations are described. PRP should be prepared as soon as possible after blood collection, to avoid undesired platelet activation [46]. It is recommended activating platelets by adding CaCl_2_ [43] and a basic substance like NaHCO_3_ to get a pH greater than 8.0 that stimulates platelet activation [46]. Double freeze thaw technique was compared to CaCl_2_ as a growth factor concentration technique [53]. Levels of PDGF-AB and PDGF-BB were significantly higher with CaCl_2_ technique whereas there was a nonsignificant trend to higher levels of TGF-*β*1 and IGF-1 with double-thaw technique. The exact platelet count and RBC and WBC count are still unknown. The potential effect of the volume of PRP also has to be considered [55].

A standardization of PRP preparation technique and the use of a reproducible technique would be of great help to interpret and compare the results of the numerous studies, to secondarily bring out the characteristics of the PRP which gives the best results. This current lack and important variability of PRP make this task in time confusing. The degree of knee OA also influences the efficacy of PRP injection and duration of symptoms relief. Better results are achieved in young patients with a little cartilage degeneration [14, 17, 32]. Kon et al. compared the efficacy of PRP injection in patients affected by cartilage degenerative lesions and early and severe knee OA. Better results were achieved in younger patients with a low degree of cartilage degeneration [33].

Limitations of our study are the following. We did not perform a new systematic review of the literature, because many recent meta-analyses are available. We did not compare the studies' methodology, which is very miscellaneous. We arbitrarily decided to be more discriminant in using the cut-off score of twice the MCII value promoted by Tubach et al. to define the very good responders.

## 5. Conclusion

There is a lack of standardization in PRP preparation technique. Our study helped identify features of PRP recommended for knee OA treatment, such as the use of a single spinning technique, a platelet concentration lower than 5 times the baseline (from 3 to 4), and avoiding RBC and WBC. We recommend leveraging this information about PRP for future studies.

## Figures and Tables

**Table:** (a) PAW classification system

(i) Platelet concentration	P1: ≤baseline
	P2: >baseline–750000/*µ*l
	P3: >750000–1250000/*µ*l
	P4: >1250000/*µ*l

(ii) X	Exogenous activation

(iii) White blood cells	(i) Total WBCs
	A: above baseline
	B: ≤baseline
	(ii) Neutrophils:
	A: above baseline
	B: ≤baseline

**Table:** (b) Mishra classification

	WBC	Activation?	Platelet concentration
Type 1	Increased	No	A: ≥5x
			B: <5x
Type 2	Increased	Yes	A: ≥5x
			B: <5x
Type 3	Minimal or no WBCs	No	A: ≥5x
			B: <5x
Type 4	Minimal or no WBCs	Yes	A: ≥5x
			B: <5x

**Table 2:** The outcomes assessed and reported by each author. K-L score = Kellgren-Lawrence classification score; ref = references; stage = stage of osteoarthritis; VAS = visual analogic scale; + = >2xMCII (VGRG); 0 = 2xMCII > 0 > 1xMCII; − = <MCII (BRG).

Ref	Stage	WOMAC	VAS	IKDC	KOOS	Efficacy
Vaquerizo et al. [25]	K-L score: 2 to 4	WOMAC total score: change from baseline −42% at 24 weeks, −34% at 48 weeks	—	—		+

Patel et al. [26]	Ahlbäck grade 1 or 2 (3) without deformation	WOMAC total score: change at 6 months: −51% (1 inj PRP), −43% (2 inj PRP)				+

Filardo et al. [27]	K-L score: 0 to 3				No improvement >15 in absolute value at 6 and 12 months and no statistically significant difference with HA	−

Cerza et al. [28]	K-L score: 1, 2, 3	WOMAC score before treatment: 79,6; 24 weeks: 36,5; difference: −43,1				+

Sánchez et al. [29]	Ahlbäck grade < 4	WOMAC total score improvement: 35,1%				0

Say et al. [30]	K-L score: 1, 2, 3				KOOS 46 at baseline; 84,4 at 6 months, +38,4/100, absolute value	+

Spaková et al. [31]	K-L score: 1, 2, 3	WOMAC OA index: 38,76 at baseline; 14,35 at 3 months; improvement: 24,41		—		0

Filardo et al. [32]	K-L score: 0; 1 to 3; 4	—		IKDC from 45.0 at baseline to 61,3 at 6 months in PRGF group (increase 16,3) and from 42,1 at baseline to 62,5 at 6 months in PRP group (increase 20,4)		0

Kon et al. [33]	K-L score: 0; 1 to 3; 4	—	53,6 at baseline to 72,3 at 6 months (18,7)	IKDC: 41,2 at baseline; 64,00 at 6 months (increase 22,8)		0

Acosta-Olivo et al. [20]	K-L score: 1	—			KOOS: from 30,1 to 48.2 (60%)	Relative improvement: very good (not for absolute value)

Kon et al. [34]	K-L score: 0, 1–3, 4	—	Basal 50.9 = chondropathy; 51.8 = early OA; 50.3 = advanced OA 2 months 75.6–71.6–14.9 60.0–16.4 6 months 78.0–67.5–57.1 12 months 78.9–69.2–56.9	Basal 43.1–40.0–34.9 2 months 70.1–59.5–48.1 6 months 72.4–58.9–44.2 12 months 68.7–56.8–46.4		0

Filardo et al. [35]	K-L score: 0, 1–3, 4	—	Follow-up of KON at 24 months: 50 to 70 at 12 months to 60 at 24 months	The IKDC objective score increased from 47% of normal and nearly normal knees before the treatment to 78% at the end, 73 and 67% at the 6- and 12-month follow-ups. The evaluation after 24 months showed a drop (*P* = 0.04) in the score to 59%. IKDC subj: 40 to 60 at 12 months, 51 at 24 months		0

Sampson et al. [36]	History of gonarthrosis, primary or secondary for more than 3 months	—	Rest: 2.5 at baseline; 0.8 at 52 weeks Moving: 4.6 at baseline; 2.5 at 52 weeks Bent knee: 2.8 at baseline; 1.3 at 52 weeks	—	Pain: 35.3–48.1 (12.8) Symptom relief: 36.1–43.9 (7.8) Activity of daily living: 44.8–54.3 (9.5) Sports: 9.6–20 (10.4) Quality of life: 1.0–13.4 (12.4)	−

Wang-Saegusa et al. [37]	Outer bridge grades I–IV (MRI)	The patients follow-up improvement was 65.5, 48.2, and 67.4% for pain, stiffness, and functional capacity, respectively	The VAS score for pain was 4.86 before treatment and 3.32 follow-up	—	—	+

Gobbi et al. [38]	K-L score: 1 to 3	—	Before 6 months–12 months: 4,1–2,2–1,2	IKDC (PRE-6 months–12 months) 48.2–65.2–75.4	KOOS Pain 73.6 ± 4.3 81.9 ± 4.3 88.7 ± 2.9 32.333 Symptoms 72.0 ± 4.1 78.2 ± 4.2 86.4 ± 3.2 27.674 Activities of daily living 77.8 ± 5.7 86.3 ± 4.7 94.8 ± 2.5 19.163 Sport 42.3 ± 7.3 50.6 ± 7.6 63.8 ± 6.7 22.176 Quality of life 41.3 ± 5.3 52.5 ± 5.2 68.0 ± 5.6 43.305	0

Napolitano et al. [39]	K-L score: 1 to 3	Arthritis: (A) pain, before treatment 10.4 ± 3.9, after treatment 17 ± 2.5, and, for 7 patients, at the 6-month follow-up 17.9 ± 2.8; (B) joint stiffness, before treatment 4.9 ± 2.2, after treatment 7 ± 0.9, and at the 6-month follow-up 7.4 ± 0.9; (C) function, before treatment 36.3 ± 11.8, after treatment 58.9 ± 9.9, and at the 6-month follow-up 60.7 ± 7.6 Cartilage disease: (A) pain, before treatment 13.0 ± 4.8, after treatment 18 ± 2.5 and, for 6 patients, at the 6-month follow-up 18 ± 2.8; (B) joint stiffness, before treatment 5.1 ± 2.2, after treatment 6.8 ± 1.0, and at the 6-month follow-up 6.8 ± 1.3; (C) function, before treatment 46.2 ± 13.1, after treatment				−

Halpern et al. [40]	K-L score: 0, 2	WOMAC total 45,1% change from baseline at 6 months; 56,2% at 12 months	VAS pain at 6 months 56,2% change from baseline; 58,9% at 12 months			+

Jang et al. [41]	K-L score: 1 to 3		VAS score improved from 7.4 before the procedure to 5.0, 4.5, and 4.2 for 1, 3, and 6 months after procedure	IKDC score changed from 54.1 before the procedure to 53.9 at 1 month after procedure, 61.6 at 6 months after procedure, and 50.3 at 1 year after procedure	—	0

Filardo et al. [21]	K-L score: 0 to 3			IKDC subjective score: 52.4 at baseline; 65.0 at 6 months; 66.2 at 12 months	KOOS score: (baseline – 6 months (diff)) Symptom 65.5–74.7 (9.2) Pain: 66.1–74.7 (8.6) ADL: 70.6–79.1 (8.5) Sport: 37.9–49.6 (11.7) QOL: 36.0–49.2 (13.2)	−

**(a):** 

Ref	Inj	Inter	Vol	Tech	Speed	Num	Time	Anticoag	Concentr
Vaquerizo et al. [25]	3	2	8	Endoret	If like Sanchez: 580 g/8 min (=1861 rpm if *r* = 15 cm)	1	8 min centrifugation + 20 min preparation	3,8% citrate	<5x baseline (via Laudy), value NM

Patel et al. [26]	1 or 2	3	8	Centrifugation: BIOAIR Safe flow 1.2, Euroclone, Siziano, Italy	1500 rpm, 15 min	1	1 to 2 h (30 min preparation)	CPDA 1 = citrate, phosphate, dextrose, adenine	310.14 × 10^3^/mL

Cerza et al. [28]	4	1	5.5	ACP	NM, Arthrex brochure: 1500 rpm, 5 min	1	NM	1 ml sodium citrate	>5x baseline (via Laudy), value NM (2 to 3 times baseline, brochure Arthrex)

Say et al. [30]	1	—	2.5	Endoret	1800 rpm, 8 min	1	30 min	3,2% sodium citrate	4x baseline, 818 520 mean value

Acosta-Olivo et al. [20]	2	2	5	GPS III Platelet Concentrate Separation Kit with ACD-A (Biomet, USA)	NM	NM	NM	NM	NM

Wang-Saegusa et al. [37]	3	2	5	Endroret	1800 rpm, 8 min	1	NM	Sodium citrate	NM

Halpern et al. [40]	1	—	6	MTF Cascade system	NM	NM	NM	NM	NM

Filardo et al. [27]	3	1	5	Double centrifugation	1480 rpm, 6 min and then 2 3400, 15 min	2	NM	NM	5x baseline (via Laudy), value NM

Sampson et al. [36]	3	4	6	GPS III Platelet Concentration System (Biomet Biologics, Warsaw, IN)	15 mins at 1700 g (Model 755 VES, The Drucker Company, Philipsburg, PA) = 3185 rpm if *r* = 15 cm	NM	NM	6 ml citrate dextrose	NM

Napolitano et al. [39]	3	1	5	Specific Fibrin Polymer 2 test-tube from RegenLab®, simple centrifugation	3100 rpm, 8 min	1	NM	NM	Platelet concentration factor: 2.3 (minimum 0.17, maximum 5.23)

Filardo et al. [21]	3	1	5	Double centrifugation	1480, 6 min and then 3400, 15 min	2	NM	NM	4.6 ± 1.4 times

**(b):** 

Ref	RBC	WBC	Activ	Tampon	Rehab	Mish	PAW	Storage
Vaquerizo et al. [25]	NM	No	yes: CaCl_2_ 400 *µ*l	NM	NM	4B	P2B*β* (Delong et al.)	Blood extracted each time

Patel et al. [26]	No	No	1 ml CaCl_2_ for 4 ml prp	NM	Mobilisation 5–10 min then SLR, static quads, hamstring reinforcement	4B	P2B*β*	Blood extracted each time

Cerza et al. [28]	NM	None	No	NM	No	3A	P2B*β* (Delong et al.)	Blood extracted each time

Say et al. [30]	No	None or a few	yes: 50 *µ*l CaCl_2_ for 1 ml prp = 5,5%	NM	Rest, paracetamol and cryotherapy, no NSAIDs	4B	P2B*β* (Delong et al.)	—

Acosta-Olivo et al. [20]	NM	NM	NM	NM	Relative rest 24 h, physical therapy: isometric and isotonic contractions	3A (Kaux et al.)	P2-4A*α* (Delong et al.)	Blood extracted each time

Wang-Saegusa et al. [37]	NM	No	10% CaCl_2_	NM	NM	4B (Endoret)	P2B*β* (Delong et al.)	Not mentioned, freeze?

Halpern et al. [40]	NM	NM	NM	NM	NM	3 or 4B (activation?) (Delong et al.)	P2B*β* (Delong et al.)	—

Filardo et al. [27]	NM	Yes: 1,2x baseline	NM	NM	Rest, light activity	2A	P3Ax	Freeze −30°

Sampson et al. [36]	NM	NM	6 ml prp + 0.6 ml of a 1000 U/ml bovine thrombin suspension in 10% calcium chloride solution	NM	Passive flexion and extension, 3 times, and then 10 mins of rest, acetaminophen and hydrocodone against pain, relative rest 24 H after injection, no standardised protocol	3A (Kaux et al.)	P2-4A*α* (Delong et al.)	Blood extracted each time

Napolitano et al. [39]	No	NM	Calcium gluconate 10%	NM	No NSAIDs, no heavy physical activity for lower limbs for 2 weeks	2B (Magalon et al.)	P2Ax (Magalon et al.)	Blood extracted each time

Filardo et al. [21]	NM	Yes: 1,1 ± 0,5	10% CaCl_2_	NM	NM	2B	P3Ax	Freeze −30°
